# Association between diabetes mellitus, prediabetes and risk, disease progression of Parkinson's disease: A systematic review and meta-analysis

**DOI:** 10.3389/fnagi.2023.1109914

**Published:** 2023-03-16

**Authors:** Qifan Zhong, Shenglong Wang

**Affiliations:** Department of Neurology, The Affiliated Jiangsu Shengze Hospital of Nanjing Medical University, Suzhou, Jiangsu, China

**Keywords:** diabetes mellitus, meta-analysis, Parkinson's disease, prediabetes, risk

## Abstract

**Background:**

Previous studies reported inconsistent results regarding association between diabetes mellitus (DM), prediabetes and risk, disease progression of Parkinson's disease (PD). The meta-analysis was made to investigate association between DM, prediabetes and risk, disease progression of PD.

**Methods:**

Literatures investigating association between DM, prediabetes and risk, disease progression of PD were searched in these databases: PubMed and Web of Science. Included literatures were published before October 2022. STATA 12.0 software was used to compute odds ratios (ORs)/relative risks (RRs) or standard mean differences (SMDs).

**Results:**

DM was associated with a higher risk of PD, compared to non-diabetic participants with a random effects model (OR/RR = 1.23, 95% CI 1.12–1.35, *I*^2^ = 90.4%, *p* < 0.001). PD with DM (PD-DM) was associated with a faster motor progression compared to PD without DM (PD-noDM) with a fixed effects model (RR = 1.85, 95% CI 1.47–2.34, *I*^2^ = 47.3%, *p* = 0.091). However, meta-analysis for comparison in change rate of United Rating Scale (UPDRS) III scores from baseline to follow-up time between PD-DM and PD-noDM reported no difference in motor progression between PD-DM and PD-noDM with a random effects model (SMD = 2.58, 95% CI = −3.11 to 8.27, *I*^2^ = 99.9%, *p* < 0.001). PD-DM was associated with a faster cognitive decline compared to PD-noDM with a fixed effects model (OR/RR = 1.92, 95% CI 1.45–2.55, *I*^2^ = 50.3%, *p* = 0.110).

**Conclusions:**

In conclusion, DM was associated with a higher risk and faster disease decline of PD. More large-scale cohort studies should be adopted to evaluate the association between DM, prediabetes and PD.

## Introduction

Diabetes mellitus (DM) has been deemed as one of the most common and serious chronic diseases worldwide, resulting in disabling, life threatening and costly complications, and eventually shortening life expectancy, reducing the quality of life (Heald et al., 2020). DM has a global prevalence of 9% (463 million adults) in 2019 (Sun et al., 2022). In addition, the global prevalence of DM is rising due to the aging of populations. Prediabetes, a high-risk period for DM, is defined by the blood glucose levels between normal and diabetes thresholds. Five to ten percent of prediabetes patients progress into patients with DM per year (Tabák et al., 2012).

Parkinson's disease (PD) is the second most common neurodegenerative disease and affects more than 1% of the population with the age of > 50 years old (Calabrese, 2007). Some studies (Deischinger et al., 2021; Sánchez-Gómez et al., 2021) reported that DM had a higher risk to be diagnosed with PD compared to non-diabetic participants, whereas some studies (Skeie et al., 2013; De Pablo-Fernandez et al., 2017) did not show any association between prevalence of PD and DM. In addition, some studies (Athauda et al., 2022) found that PD with DM (PD-DM) patients had significantly faster motor symptom progression and were more likely to develop mild cognitive impairment compared with PD without DM (PD-noDM), whereas some studies (Ou et al., 2021) reported no significant association between DM and disease progression of PD. In addition, no sufficient information was supported for association between prediabetes and PD. The present meta-analysis was made to investigate the association between DM, prediabetes and risk, disease progression of PD.

## Methods

The study was performed according to the Preferred Reporting Items for Systematic reviews and Meta-Analysis (PRISMA) guideline (Moher et al., 2009). Supplementary Table 1 showed the PRISMA checklist.

### Search strategy

Literatures investigating association between DM, prediabetes and risk, disease progression of PD were searched in these databases: PubMed and Web of Science. Included literatures were published before October 2022. We used these search terms: (“diabetes” OR “prediabetes” OR “glucose” OR “hyperglycemia” OR “insulin resistance” OR “HbA1_c_”) AND (“Parkinson's disease” OR “Parkinson's disease”).

### Inclusion criteria and exclusion criteria

We adopted these inclusion criteria: (1) studies investigated DM or prediabetes; (2) studies investigated PD; (3) studies were published in English. We adopted these exclusion criteria included: (1) reviews, meta-analysis and case-reports were excluded; (2) literatures were excluded if literature did not provide sufficient information for odds ratios (ORs)/relative risks (RRs) and 95% confidence intervals (CIs) regarding association between DM, prediabetes and risk, disease progression of PD.

### Data extraction and meta-analysis

These data were extracted from included literatures with Excel document: Author and publication year, study location, study type, sample size, age, gender, event for analysis, results and adjusted variables.

We adopted STATA 12.0 software to compute the results. ORs/RRs and 95% CIs were computed to acquire a computed OR/RR and 95% CI. In addition, a computed standard mean difference (SMD) and a 95% CI was acquired using STATA 12.0 software. *P*-value < 0.05 was considered statistically significant. A random effects model was used for high heterogeneity (*p*-value for *Q*-test ≤ 0.05 and *I*^2^ ≥ 50%); inversely, a fixed effects model was used for low heterogeneity (*p*-value for *Q*-test > 0.05 and *I*^2^ <50%). We adopted meta-regression analysis and subgroup studies (for different ethnicities and different study types) to investigate the source of heterogeneity. Sensitivity analysis was employed to assess the study stabilization. We adopted Begg's test, Egger's test and funnel plot to evaluate publication bias. Quality appraisal was conducted using the Cochrane Risk of Bias Tool. Data were analyzed with Review Manager 5.3.

## Results

### Characteristics regarding included studies

**Figure 1:**
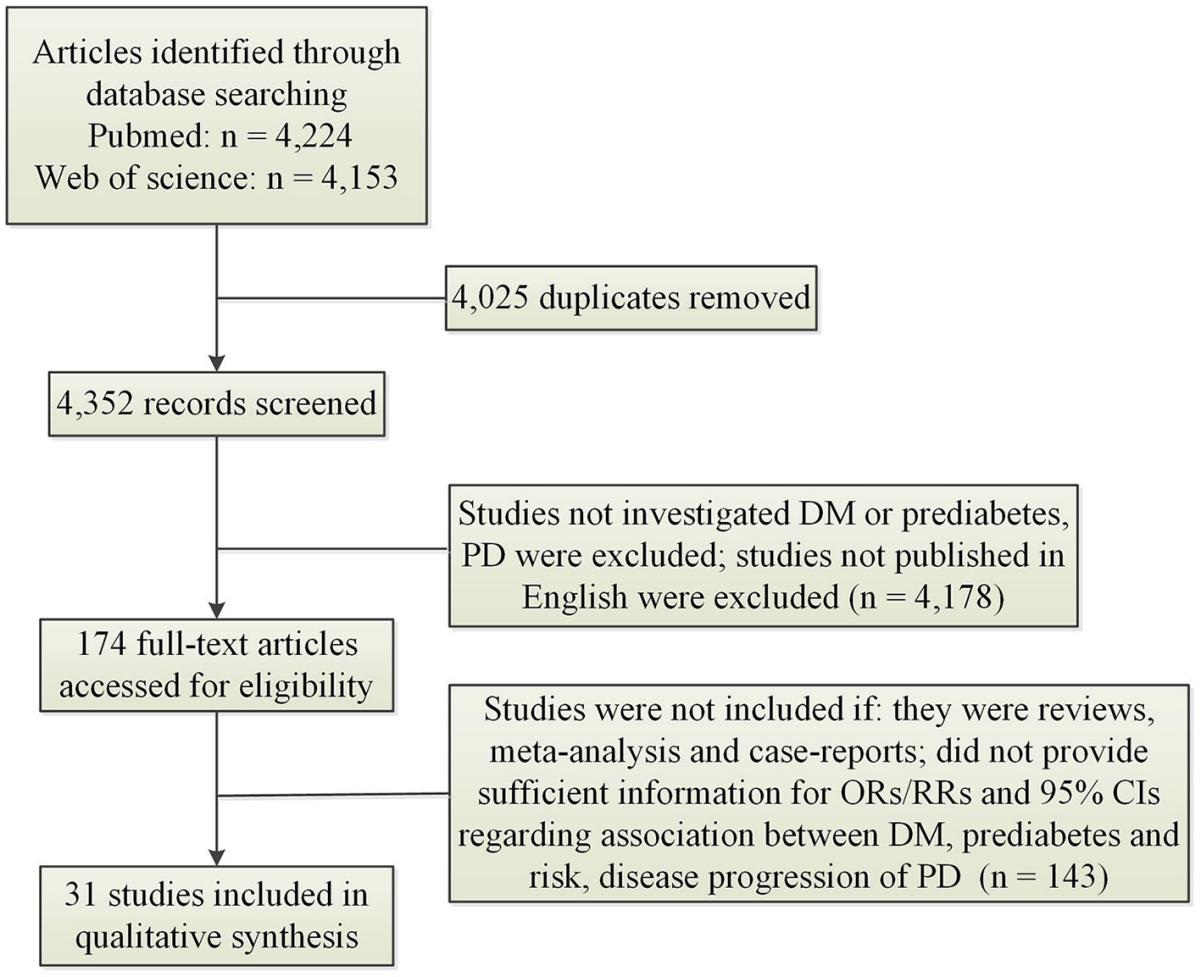
Search results and selection process. CI, confidence interval; DM, diabetes mellitus; OR, odds ratio; PD, Parkinson's disease; RR, relative risk.

Figure 1 illustrated result of initial search and study selection process. Tables 1, 2 showed characteristics regarding included studies. *N* = 12 case-control studies (Morano et al., 1994; Leibson et al., 2006; Powers et al., 2006; Scigliano et al., 2006; Becker et al., 2008; D'Amelio et al., 2009; Rugbjerg et al., 2009; Miyake et al., 2010; Schernhammer et al., 2011; Savica et al., 2012; Skeie et al., 2013; De Pablo-Fernandez et al., 2017) [including *N* = 21,118 PD patients and *N* = 89,150 healthy controls (HCs)], *N* = 11 cohort studies (Grandinetti et al., 1994; Hu et al., 2007; Simon et al., 2007; Driver et al., 2008; Palacios et al., 2011; Xu et al., 2011; Sun et al., 2012; Yang et al., 2017; De Pablo-Fernandez et al., 2018; Kizza et al., 2019; Sánchez-Gómez et al., 2021) (including *N* = 35,939 PD patients and *N* = 7,062,700 participants) and *N* = 1 cross-sectional study (Deischinger et al., 2021) (including *N* = 235,268 PD patients and *N* = 1,938,173 HCs) were included regarding association between DM and risk of PD. Only *N* = 1 cohort study (Sánchez-Gómez et al., 2021) (including *N* = 13,715 PD patients and *N* = 3,104,460 participants) was included for association between prediabetes and risk of PD. *N* = 7 cohort studies (Cereda et al., 2012; Malek et al., 2016; Ong et al., 2017; Pagano et al., 2018; De Pablo-Fernandez et al., 2021; Ou et al., 2021; Athauda et al., 2022) (including *N* = 473 PD-DM patients and *N* = 4,081 PD-noDM patients) were included regarding association between DM and progression of PD.

**Table 1:** Characteristics of included studies exploring association between DM, prediabetes and risk of PD.

**References**	**Study location**	**Study design**	**Sample size**	**Age (years)**	**Gender (male%)**	**DM or prediabetes**	**Results (OR/RR, 95% CI)**	**Adjustment factor**
Sánchez-Gómez et al. (2021)	Spain	Cohort	13,715PD/3,104,460	NR	48.2%	DM, prediabetes	DM: RR: 2.36 (1.96–2.84); Prediabetes: RR: 2.10 (1.70–2.59)	Age and sex, BMI, smoking status and socioeconomic status
Deischinger et al. (2021)	Austria	Cross-sectional	235,268PD/1,938,173	64.36 ± 10.03	60.79%	DM	OR: 1.46 (1.38–1.54)	NR
Kizza et al. (2019)	China	Cohort	603PD/503,497	30–79 years	40.8%	DM	RR: 0.93 (0.67, 1.29)	Age-at-risk, region, income, education, occupation, alcohol consumption, and physical activity
De Pablo-Fernandez et al. (2018)	UK	Cohort	14,252 PD/2,017,115	NR	61.1%	DM	RR: 1.32 (1.29, 1.35)	Age, sex, calendar year of cohort entry, region of residence, and patients' quintile of Index of Multiple Deprivation score (a measure of area-level deprivation)
De Pablo-Fernandez et al. (2017)	Spain	Case-control	79PD/4,998 controls	73	42.3%	DM	OR: 1.89 (0.90, 3.98)	Sex, age, hypertension, dyslipidaemia, antidiabetic treatment, alcohol consumption, smoking status, body mass index, presence of cerebrovascular disease and treatment with potential parkinsonism-inducing drugs
Yang et al. (2017)	China, Taiwan	Cohort	1782PD/145,176	56.21 ± 13.74	53.4%	DM	RR: 1.19 (1.08, 1.32)	Age, gender, insurance premium, urbanization level, residential area, type of occupation, comorbidity, CCI score, flunarizine use, metoclopramide use, zolpidem use, and outpatients claim times
Skeie et al. (2013)	Norway	Case-control	212PD/175	NR	NR	DM	OR: 1.94 (0.82–4.57)	NR
Savica et al. (2012)	USA	Case-control	196 PD/196	71	61.7%	DM	OR: 0.67 (0.31–1.48)	Age and sex
Sun et al. (2012)	China, Taiwan	Cohort	1,613 PD/603,416	NR	49.4%	DM	RR: 1.61 (1.56, 1.66)	Age, sex, geographic area, urbanization status, hypertension, hyperlipidemia, and cardiovascular disease
Schernhammer et al. (2011)	USA	Case-control	1,931 PD/9,651	72.2 ± 10.5	58.1%	DM	OR 1.36 (1.08–1.71)	Age, sex, and chronic obstructive pulmonary disease
Palacios et al. (2011)	USA	Cohort	656 PD/147,096	71.9	43.0%	DM	RR: 0.88 (0.62, 1.25)	Age, smoking, alcohol intake, caffeine intake, calories, dairy intake, pesticide exposure, physical activity, and education
Xu et al. (2011)	USA	Cohort	1,565 PD/288,662	66.7 ± 7.3	58.2%	DM	RR: 1.41 (1.20, 1.66)	Age, sex, race, education, smoking, coffee, BMI, and physical activity
Miyake et al. (2010)	Japan	Case-control	249PD/368	68.5 (8.6)	37.9%	DM	OR: 0.38 (0.17, 0.79)	Sex, age, region of residence, pack-years of smoking, years of education, leisure-time exercise, body mass index, dietary intake of energy, cholesterol, vitamin E, alcohol, and coffee and the dietary glycemic index
Rugbjerg et al. (2009)	Denmark	Case-control	13,695 PD/68,445	73.0	NR	DM	OR: 1.1 (0.8–1.5)	COPD
D'Amelio et al. (2009)	Italy	Case-control	318PD/318	53.5	48.1%	DM	OR: 0.4 (0.2, 0.8)	Gender, age at PD onset, BMI, smoking habit, alcohol, and coffee consumption
Becker et al. (2008)	UK	Case-control	3,637 PD/3,637	60	60%	DM	OR: 0.95 (0.80, 1.14)	BMI, smoking, asthma/COPD, dementia, hypertension, ischemic heart disease, congestive heart failure, stroke/transient ischemic attack, arrhythmia, hyperlipidemia, epilepsy, affective disorders, schizophrenia, and neurotic and somatoform disorders
Driver et al. (2008)	USA	Cohort	556 PD/21,841	NR	100%	DM	RR: 1.34 (1.01, 1.77)	Age, smoking status, alcohol use, BMI, physical activity vigorous enough to work up a sweat, hypertension, and cholesterol levels
Hu et al. (2007)	Finland	Cohort	609 PD/51,552	53.3 (10.6)	48.8%	DM	RR: 1.83 (1.21, 2.76)	Age, study year, BMI, systolic blood pressure, cholesterol, education, leisure-time physical activity, cigarette smoking, coffee consumption, tea consumption, and alcohol consumption
Simon et al. (2007)	USA	Cohort	530 PD/171,879	45.3	29.6%	DM	RR: 1.04 (0.74, 1.46)	Age and smoking status
Powers et al. (2006)	USA	Case-control	352PD/484	69	61.6%	DM	OR: 0.62 (0.38, 1.01)	Age, ethnicity, education, and smoking
Scigliano et al. (2006)	Italy	Case-control	178PD/533	58.1	51.7%	DM	OR: 0.30 (0.13, 0.72)	Age and sex
Leibson et al. (2006)	USA	Case-control	197 PD/197	70 ± 11	61%	DM	OR: 0.7 (0.4–1.4)	NR
Morano et al. (1994)	Spain	Case-control	74 PD/148	NR	NR	DM	OR: 1.387 (0.570–3.261)	NR
Grandinetti et al. (1994)	USA	Cohort	58PD/8,006	NR	100%	DM	RR: 1.43 (0.82–2.52)	NR

BMI, body mass index; CI, confidence interval; COPD, chronic obstructive pulmonary disease; DM, diabetes mellitus; NR, not reported; OR, odd ratio; PD, Parkinson's disease; RR, relative risk; USA, united states; UK, united kingdom.

**Table 2:** Characteristics of included studies exploring association between DM and disease progression of PD.

**References**	**Study location**	**Study design**	**Sample size**	**Age (years)**	**Gender (male%)**	**DM or prediabetes**	**Results (RR, 95% CI)**	**Adjustment factor**
Athauda et al. (2022)	UK	Cohort	167PD-DM/1763PD-noDM	71.1 (0.7)	72.5%	DM	Faster motor progression: RR: 1.55 (1.07–2.23); Faster cognitive decline: RR: 1.74 (1.19–2.55); Baseline: UPDRS III 25.8 (0.9) vs. 22.5 (0.3); follow-up: UPDRS III 31.7 vs. 29	Age, sex, vascular score, disease duration, ethnicity, baseline LEDD, and the baseline variable value
Ou et al. (2021)	China	Cohort	49PD-DM /379 PD-noDM	68.0 (9.0)	59.2%	DM	2.060 (1.165–3.641) for UPDRS III ≥14-point increase in the poorly controlled DM group, and 1.066 (0.572–1.986) in the well-controlled DM group	Sex, age, age of onset, BMI, and UPDRS III and MoCA scores at baseline
De Pablo-Fernandez et al. (2021)	UK	Cohort	25PD-DM/107PD-noDM	70.4 ± 8.1	76%	DM	Motor progression: RR: 2.39 (1.36–4.20); cognitive decline: RR: 3.62 (1.73–7.58)	Potential confounders
Pagano et al. (2018)	UK	Cohort	25PD-DM/25PD-noDM	62.9 (9.3)	72%	DM	Faster motor progression: RR: 4.521 (1.468–13.926); Faster cognitive decline: RR: 9.314 (1.164–74.519); Baseline: UPDRS III 16.9 (6.6) vs. 24.0 (9.1); cognition: Baseline: MoCA 26.8 (2.9) vs. 26.7 (2.4)	NR
Ong et al. (2017)	Singapore	Cohort	12PD-DM/65PD-noDM	67.41 ± 4.93	NR	DM	Baseline: HandY 1.88 ± 0.43 vs. 1.91 ± 0.37; follow-up: HandY 2.05 ± 0.57 vs. 2.08 ± 0.37; cognition: Baseline: MoCA 26.75 ± 1.66 vs. 26.58 ± 3.32; decline: −3.29 ± 3.68 vs. −0.55 ± 2.48	NR
Malek et al. (2016)	UK	Cohort	106PD-DM/1,653 PD-noDM	64.3 (9.8)	65.2%	DM	Faster motor progression: RR: 3.65 (1.07, 6.22); Faster cognitive decline: RR: 1.52 (0.89, 2.58)	Age, gender, disease duration, and drug naive
Cereda et al. (2012)	Italy	Cohort	89PD-DM/89PD-noDM	70.7 (7.7)	65.1%	DM	Baseline: UPDRS III 9.7 (5.1) vs. 8.3 (4.3); follow-up: UPDRS III 22.3 (9.0) vs. 19.3 (7.9)	NR

BMI, body mass index; CI, confidence interval; DM, diabetes mellitus; HandY, Hoehn and Yahr; LEDD, levodopa-equivalent daily dose; MoCA, Montreal Cognitive Assessment; NR, not reported; PD, Parkinson's disease; RR, relative risk; UK, united kingdom; UPDRS, Unified Parkinson's disease.

### Meta-analysis results

#### Association between DM and risk of PD

**Figure 2:**
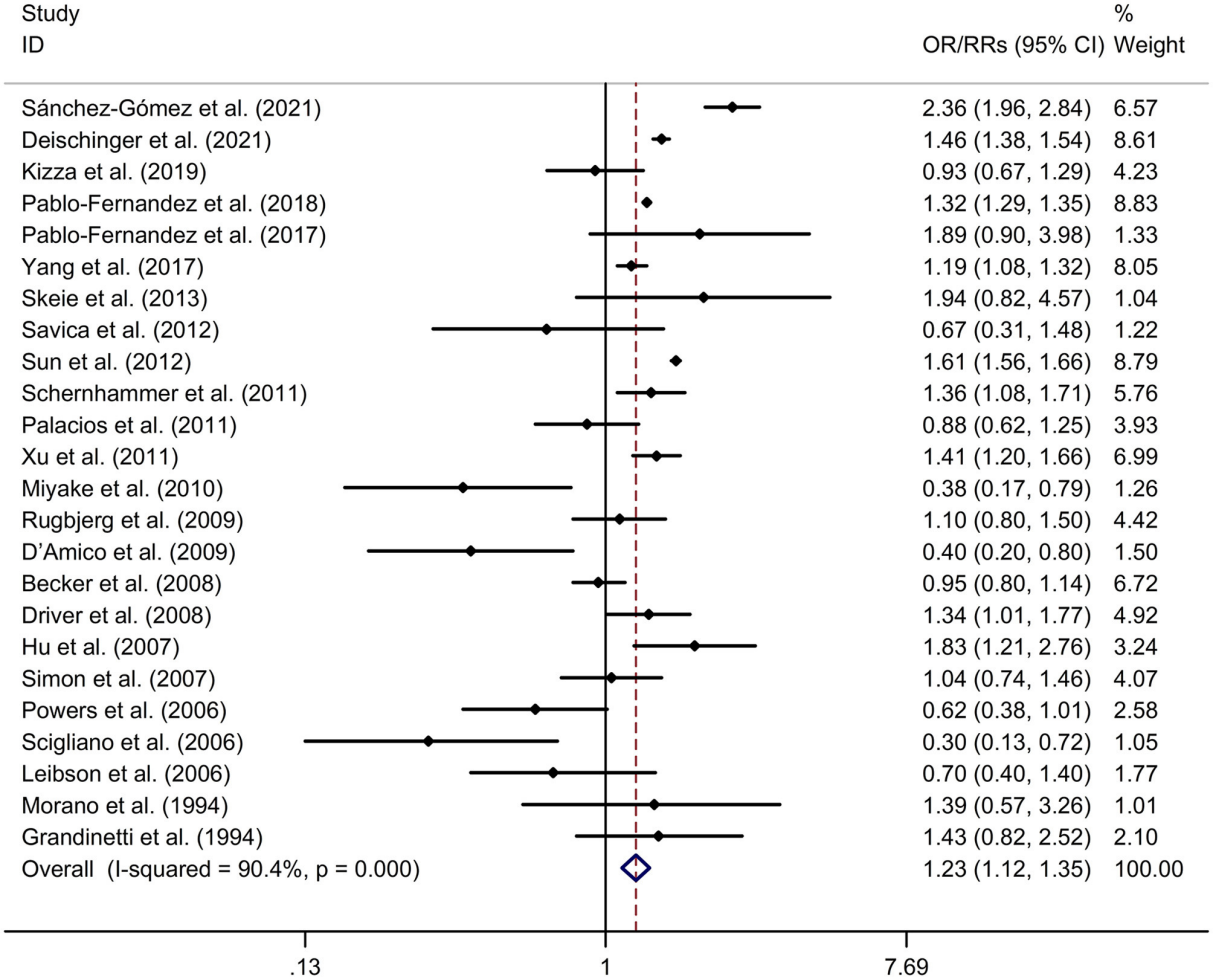
Forest plot for association between DM and risk of PD. CI, confidence interval; DM, diabetes mellitus; OR, odds ratio; PD, Parkinson's disease; RR, relative risk.

DM was associated with a higher risk of PD, compared to non-diabetic participants with a random effects model (OR/RR = 1.23, 95% CI 1.12–1.35, *p* < 0.001, *I*^2^ = 90.4%, *p*-value for *Q*-test < 0.001; Figure 2). Meta-regression analysis indicated that publication year, age and gender were not responsible for heterogeneity between studies (publication year: *p* = 0.109; age: *p* = 0.730; gender: *p* = 0.878). Subgroup analysis found that DM was associated with a higher risk of PD in Caucasian compared to non-diabetic participants, whereas no significant association was showed between DM and risk of PD in Asian (Caucasian: OR/RR = 1.23, 95% CI 1.10–1.37; Asian: OR/RR = 1.11, 95% CI 0.82–1.48; Supplementary Figure 1). Subgroup analysis found that DM was associated with a higher risk of PD in cohort studies compared to non-diabetic participants, whereas no significant association was showed between DM and risk of PD in case-control studies (cohort: RR = 1.37, 95% CI 1.23–1.54; case-control: OR = 0.86, 95% CI 0.66–1.11; Supplementary Figure 2). Sensitivity analysis indicated no change in the direction of effect while any one study was excluded from the meta-analysis (Supplementary Figure 3). Begg's test, Egger's test and funnel plot showed no significant risk of publication bias (Begg's test *p* = 0.442; Egger's test: *p* = 0.120; Supplementary Figure 4).

#### Association between DM and motor progression of PD

**Figure 3:**
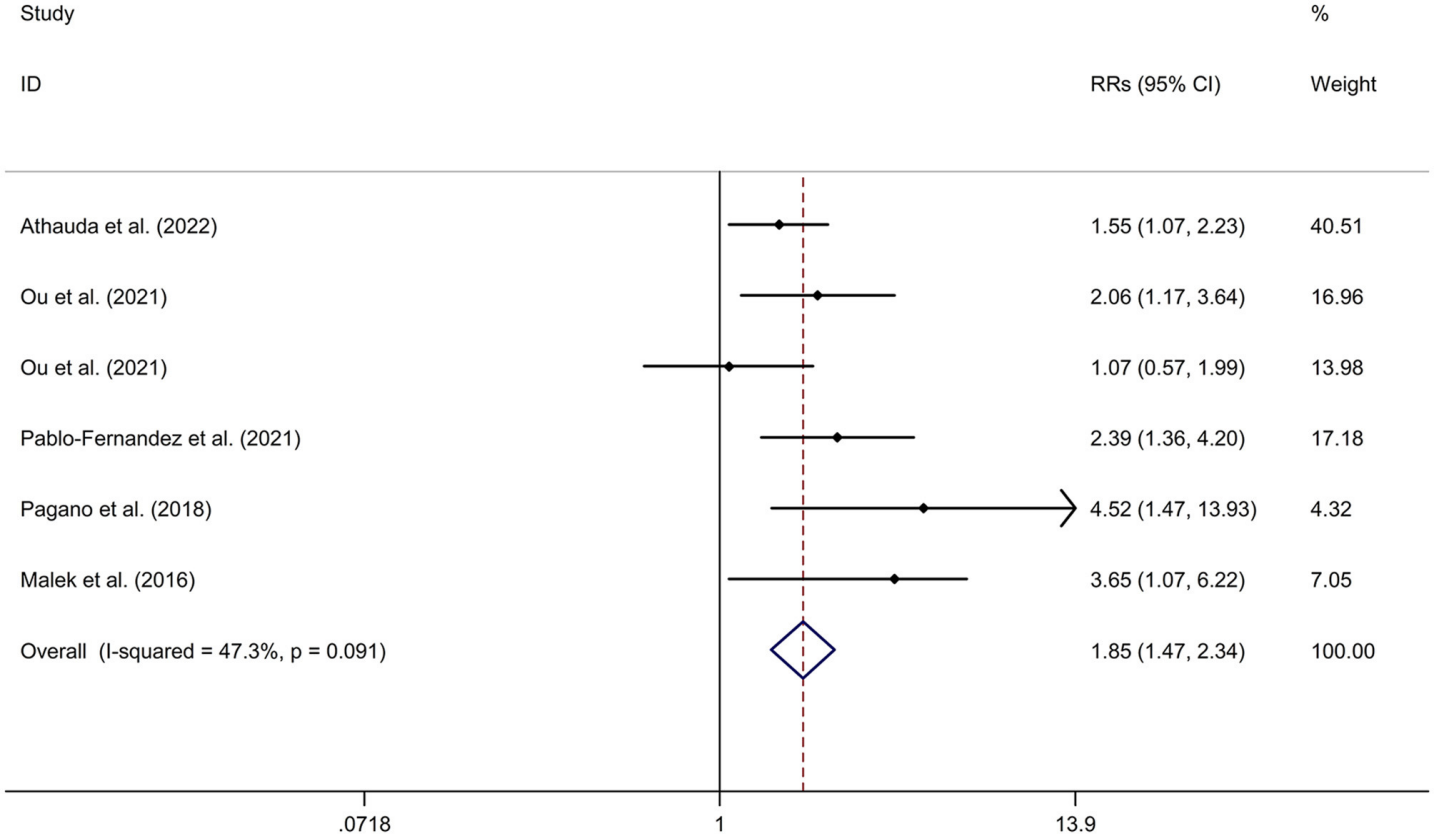
Forest plot for association between DM and motor progression of PD. CI, confidence interval; DM, diabetes mellitus; PD, Parkinson's disease; RR, relative risk.

PD-DM was associated with a faster motor progression compared to PD-noDM with a fixed effects model (RR = 1.85, 95% CI 1.47–2.34, *p* < 0.001, *I*^2^ = 47.3%, *p*-value for *Q*-test = 0.091; Figure 3). Meta-regression analysis indicated that publication year, age and gender were not responsible for heterogeneity between studies (publication year: *p* = 0.736; age: *p* = 0.652; gender: *p* = 0.371). Subgroup analysis found that PD-DM was associated with a faster motor progression in Caucasian compared to PD-noDM (OR/RR = 2.01, 95% CI 1.52–2.67; Supplementary Figure 5). Sensitivity analysis indicated no change in the direction of effect while any one study was excluded from the meta-analysis (Supplementary Figure 6). Begg's test, Egger's test and funnel plot showed no significant risk of publication bias (Begg's test *p* = 0.260; Egger's test: *p* = 0.152; Supplementary Figure 7).

**Figure 4:**
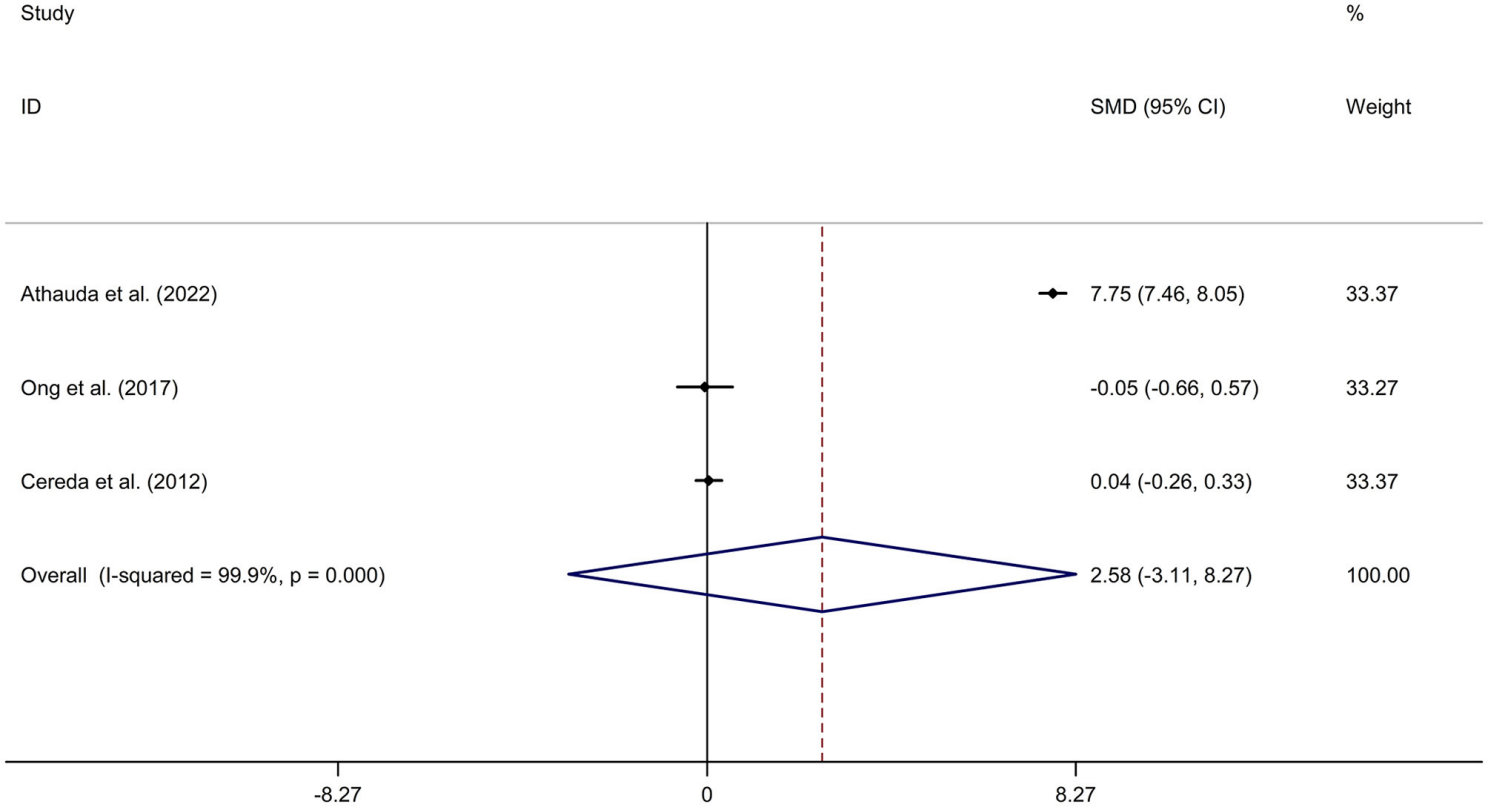
Forest plot for comparison in change of motor function between PD-DM and PD-noDM. CI, confidence interval; DM, diabetes mellitus; PD, Parkinson's disease; PD-DM, PD with DM; PD-noDM, PD without DM; SMD, standard mean difference.

Mean values and standard deviation (SD) of increase or reduction rate of United Rating Scale (UPDRS) III scores from baseline to follow-up time in PD-DM and PD-noDM were collected from studies. Meta-analysis for comparison in change rate of UPDRS III scores from baseline to follow-up time between PD-DM and PD-noDM reported no difference in motor progression between PD-DM and PD-noDM with a random effects model (SMD = 2.58, 95% CI = −3.11 to 8.27, *p* = 0.374, *I*^2^ = 99.9%, *p*-value for *Q*-test < 0.001, Figure 4). Meta-regression analysis indicated that publication year, age and gender were not responsible for heterogeneity between studies (publication year: *p* = 0.339; age: *p* = 0.598). Sensitivity analysis indicated no change in the direction of effect while any one study was excluded from the meta-analysis (Supplementary Figure 8). Begg's test, Egger's test and funnel plot showed no significant risk of publication bias (Begg's test *p* = 0.602; Egger's test: *p* = 0.792; Supplementary Figure 9).

#### Association between DM and cognitive decline of PD

**Figure 5:**
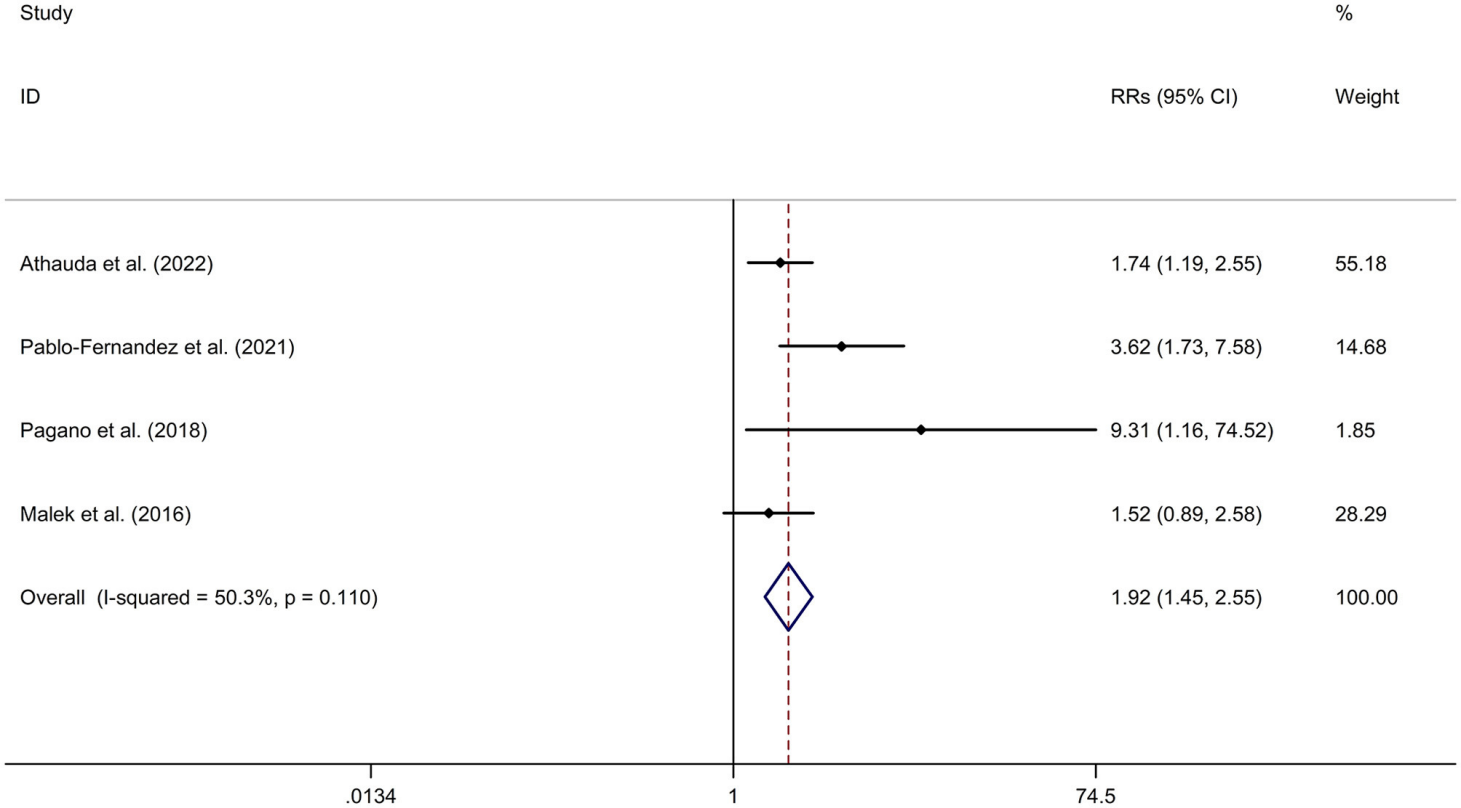
Forest plot for association between DM and cognitive decline of PD. CI, confidence interval; DM, diabetes mellitus; PD, Parkinson's disease; RR, relative risk.

PD-DM was associated with a faster cognitive decline compared to PD-noDM with a fixed effects model (OR/RR = 1.92, 95% CI 1.45–2.55, *p* < 0.001, *I*^2^ = 50.3%, *p*-value for *Q*-test = 0.110; Figure 5). Meta-regression analysis indicated that publication year, age and gender were not responsible for heterogeneity between studies (publication year: *p* = 0.477; age: *p* = 0.478; gender: *p* = 0.478). Sensitivity analysis indicated no change in the direction of effect while any one study was excluded from the meta-analysis (Supplementary Figure 10). Begg's test, Egger's test and funnel plot showed no significant risk of publication bias (Begg's test *p* = 0.497; Egger's test: *p* = 0.181; Supplementary Figure 11).

Regarding the association between prediabetes and risk of PD, Sánchez-Gómez et al. (2021) found that prediabetes were associated with a higher risk of PD (RR = 1.07, 95% CI 1.00–1.14).

Supplementary Figure 12 illustrated the risk of bias graph. Details of the risk of bias summary were showed in Supplementary Figure 13.

## Discussion

The meta-analysis found that DM was associated with a higher risk of PD, compared to non-diabetic participants. In addition, PD-DM was associated with a faster motor progression and cognitive decline, compared to PD-noDM.

Corresponding to the epidemiological evidence for the association between DM and PD, experimental research supported the common mechanisms in the two diseases. Recent evidence supported the presence of local insulin resistance in brain in neurodegenerative diseases [including PD and Alzheimer's disease (AD)] (Morris et al., 2014; Arnold et al., 2018). Brain insulin resistance refers to reaction failure of brain cells to insulin (Mielke et al., 2005). Brain insulin resistance results in deficits in neurotransmitter release or receptor regulation in neurons, neuroplasticity impairment, abnormal protein deposition and failure of clearance (Sharma et al., 2015). Additionally, systemic insulin resistance may also cause brain impairment (including inflammatory response, microvascular disease, and deficit of the blood brain barrier) through hyperglycaemia and its complications (Santiago and Potashkin, 2013). Genome-wide association studies have showed a network of genes regarding autoimmunity which is shared with PD, AD and diabetes (Menon and Farina, 2011). In addition, antidiabetic drugs have shown some immunomodulatory properties in PD animal models. Pioglitazone, a proliferator-activated receptor (PPAR)-γ agonist, can reduce microglia and astrocyte activation (Breidert et al., 2002); NLY01, a glucagon-like peptide-1 receptor (GLP1R) agonist, inhibited the phenoconversion of astrocytes toward a pro-inflammatory phenotype and protected against the loss of dopamine neurons and behavioral deficits in the model of sporadic PD (Yun et al., 2018). More studies are essential to explore the mechanism regarding association between DM and PD.

The study reported a higher risk of PD in DM. The result was consistent with a recent meta-analysis (Liu and Tang, 2021) (including 7 case-control studies and 9 cohort studies), which reported that DM was associated with an elevated risk of PD (OR/RR = 1.15, 95% CI 1.03–1.28). In addition, subgroup study was corresponding to the previous meta-analysis (Liu and Tang, 2021), which showed that DM was associated with higher risk of PD in cohort studies (RR = 1.29, 95% CI 1.15–1.45), whereas no significant association was indicated between DM and risk of PD in case-control studies (OR = 0.74, 95% CI 0.51–1.09). The different result might derive from the difference in study design. Traditionally, the results of cohort studies are usually more reliable compared to retrospective case-control studies, due to the absence of recall and interviewer bias. Subgroup analysis showed that DM was associated with a higher risk of PD in Caucasian compared to non-diabetic participants, whereas no significant association was showed between DM and risk of PD in Asian. Only *N* = 4 studies (Miyake et al., 2010; Sun et al., 2012; Yang et al., 2017; Kizza et al., 2019) explored association between DM and risk of PD in Asian. Sun et al. (2012) and Yang et al. (2017) reported that DM was associated with a significantly elevated risk of PD with cohort studies, whereas Miyake et al. (2010) reported that DM was significantly associated with a decreased risk of PD with a case-control study. The different result might derive from the difference in study design. More studies were essential to investigate association between DM and risk of PD in Asian. In addition, the result is also consistent with a recent meta-analysis (Komici et al., 2021), which found that DM patients showed a higher risk of developing PD compared to non-DM, and PD patients with DM showed a greater severity of motor symptoms, with higher motor dysfunction, compared with PD-noDM. The present meta-analysis did not explore the association between DM and disease severity of PD. Thus, more large-scale, cohort studies were needed to investigate the association between the two diseases.

PD-DM was associated with a faster motor progression and cognitive decline, compared to PD-noDM. Motor progression and cognitive decline in PD was associated with progression of dopaminergic deficit in PD (Fereshtehnejad et al., 2017). Dopamine uptake is intensive in the presence of insulin (Shaughness et al., 2020). The mechanism might mediate the association between DM and disease progression of PD. The study showed no difference in change rate of UPDRS III scores from baseline to follow-up time between PD-DM and PD-noDM. The result showed that PD-DM showed a faster motor progression, but not a greater motor progression, compared to PD-noDM. Only *N* = 3 studies were included for comparison in change rate of UPDRS III scores from baseline to follow-up time between PD-DM and PD-noDM. More cohort studies were essential to explore the association between DM and disease progression of PD.

Regarding the association between prediabetes and risk of PD, only one cohort study (Sánchez-Gómez et al., 2021) found that prediabetes were associated with a higher risk of PD. This is the first study to evaluate association between prediabetes and risk of PD development in a large cohort. A community-based study (Wong et al., 2016) reported that prediabetes was an independent risk factor of pRBD (probable rapid eye movement sleep behavior disorder), which linked prediabetes with PD. More studies are warranted to support the association between prediabetes and PD.

There are some limitations in the meta-analysis. Firstly, high heterogeneity was showed between studies exploring association between DM and risk of PD. The present meta-analysis used meta-regression analysis and subgroup analysis to investigate the source of heterogeneity across included studies. However, the source of heterogeneity still remains unclear. Secondly, the present study did not explore the association between prediabetes and PD.

## Conclusions

In conclusion, DM was associated with a higher risk and faster disease decline of PD. More large-scale cohort studies should be adopted to evaluate the association between DM, prediabetes and PD.

## Data availability statement

The original contributions presented in the study are included in the article/Supplementary material, further inquiries can be directed to the corresponding author.

## Author contributions

QZ: study design, manuscript writing, data collection, data analysis, and software use. SW: study design, manuscript writing and revision, data collection, data analysis, software use, and supervision. All authors read and approved the final version of the manuscript.
