# Supplementary material for: Association between diabetes mellitus, prediabetes and risk, disease progression of Parkinson's disease: A systematic review and meta-analysis
Source: Front Aging Neurosci. 2023 Mar 16;15:1109914. doi: 10.3389/fnagi.2023.1109914 (PMC10060805; doi:10.3389/fnagi.2023.1109914)
Supplement: Supplementary file 3 [file Table_3.DOCX]

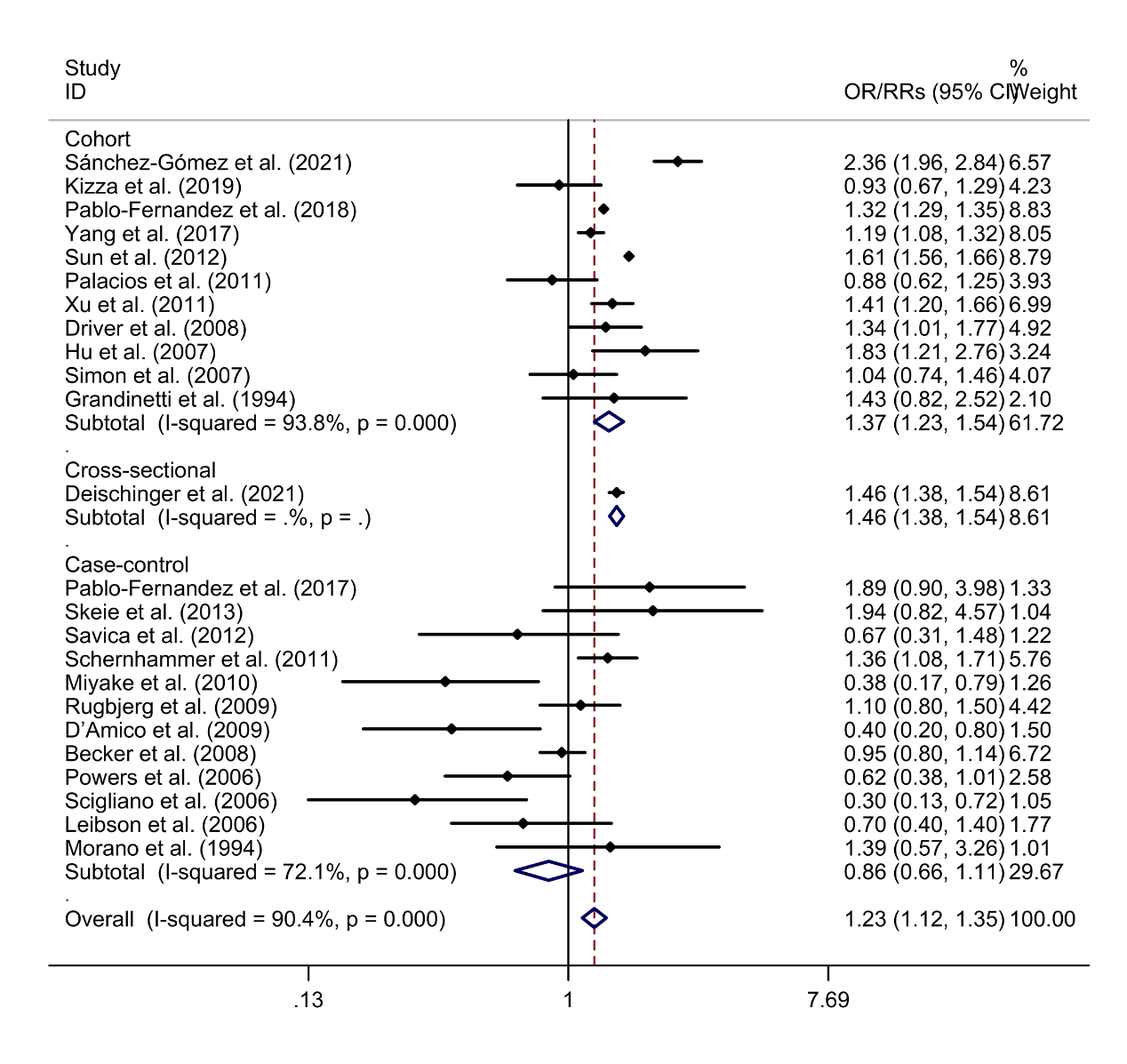


Supplementary figure 2. Subgroup analysis for association between DM and risk of PD with different study types. Abbreviations: CI, confidence interval; DM, diabetes mellitus; OR, odds ratio; PD, Parkinson’s disease; RR, relative risk.
