# Supplementary material for: Association between diabetes mellitus, prediabetes and risk, disease progression of Parkinson's disease: A systematic review and meta-analysis
Source: Front Aging Neurosci. 2023 Mar 16;15:1109914. doi: 10.3389/fnagi.2023.1109914 (PMC10060805; doi:10.3389/fnagi.2023.1109914)
Supplement: Supplementary file 6 [file Table_6.DOCX]

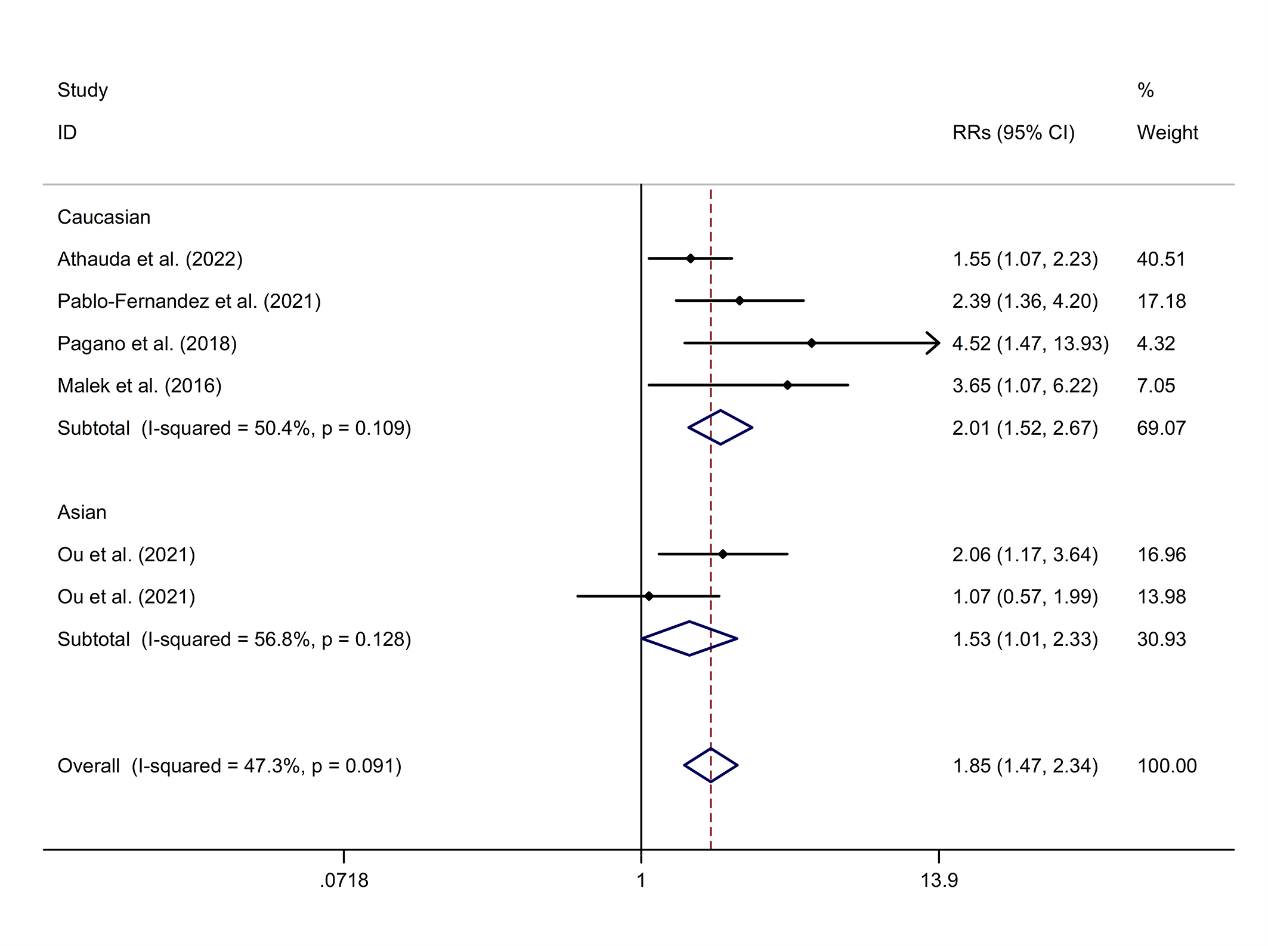


Supplementary figure 5. Subgroup analysis for association between DM and motor progression of PD with different ethnicities. Abbreviations: CI, confidence interval; DM, diabetes mellitus; PD, Parkinson’s disease; RR, relative risk.
