# Supplementary material for: Association between diabetes mellitus, prediabetes and risk, disease progression of Parkinson's disease: A systematic review and meta-analysis
Source: Front Aging Neurosci. 2023 Mar 16;15:1109914. doi: 10.3389/fnagi.2023.1109914 (PMC10060805; doi:10.3389/fnagi.2023.1109914)
Supplement: Supplementary file 8 [file Table_8.DOCX]

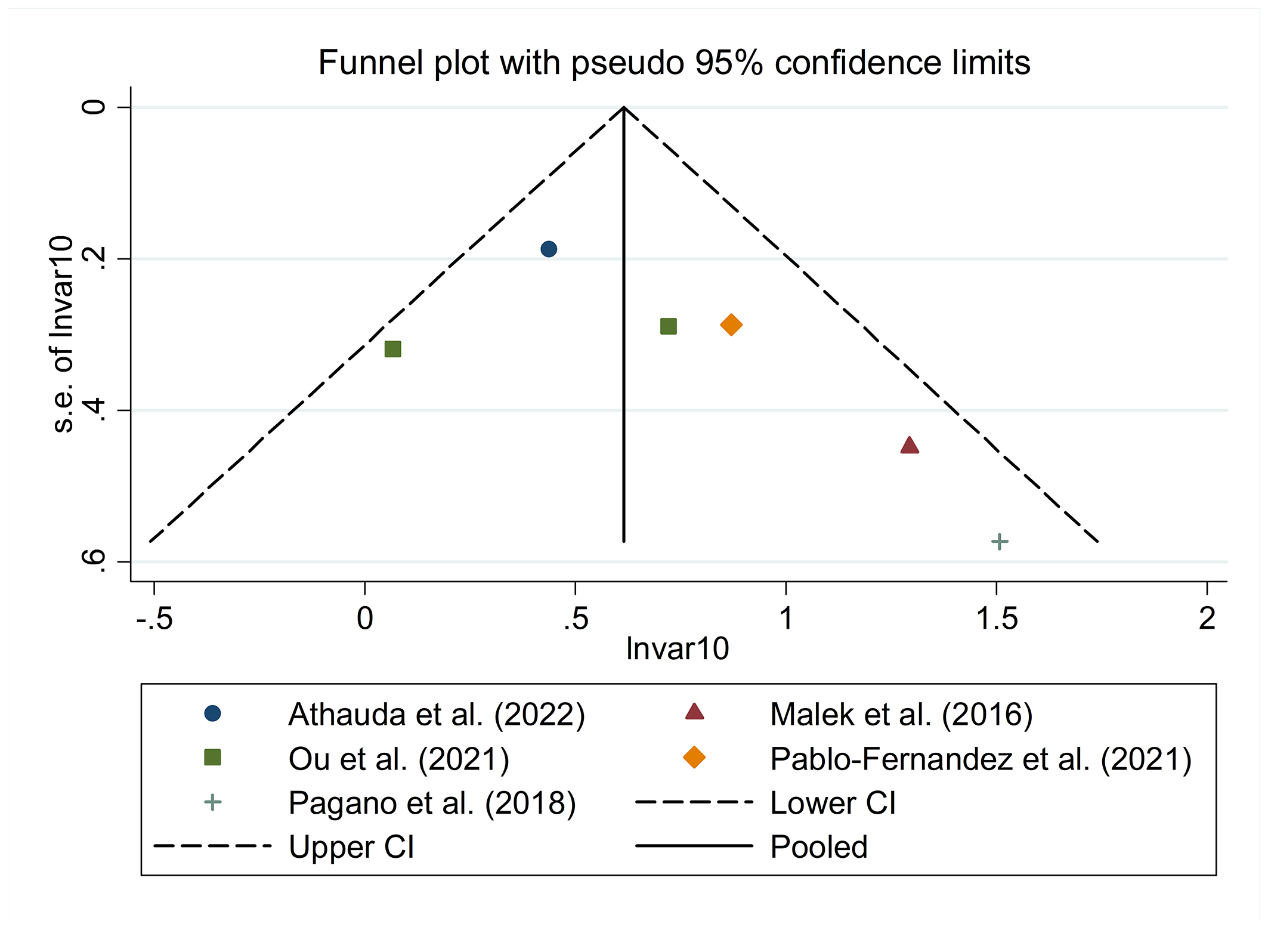


Supplementary figure 7. Funnel plot for association between DM and motor progression of PD. Abbreviations: DM, diabetes mellitus; PD, Parkinson’s disease.
