# Supplementary figures and images for: Association between diabetes mellitus, prediabetes and risk, disease progression of Parkinson's disease: A systematic review and meta-analysis
Source: Front Aging Neurosci. 2023 Mar 16;15:1109914. doi: 10.3389/fnagi.2023.1109914 (PMC10060805; doi:10.3389/fnagi.2023.1109914)

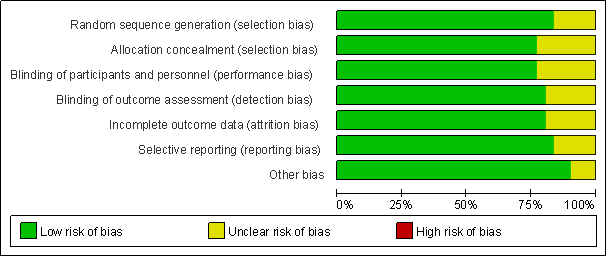


Supplementary figure 12. Risk of bias graph.

Supplement: Supplementary file 13 [file Table_13.DOCX]

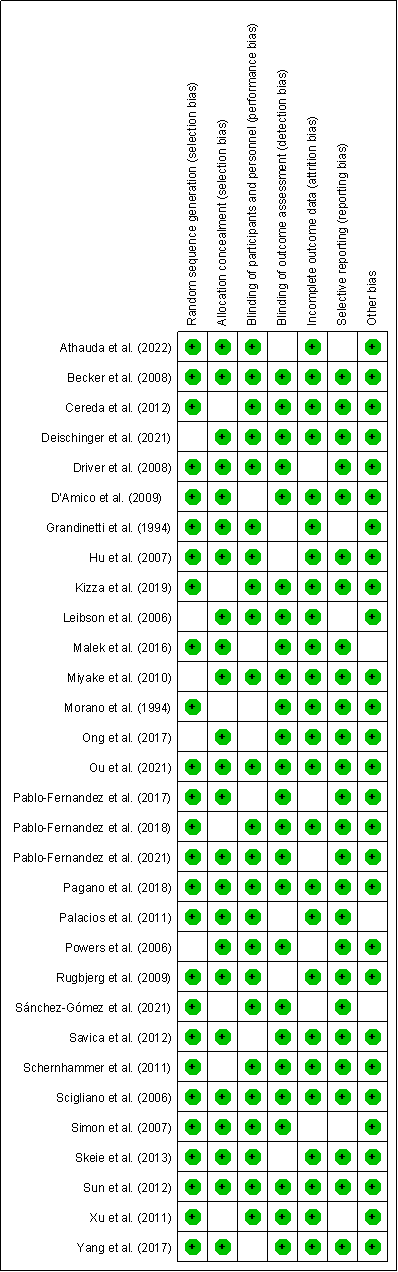


Supplementary figure 13. Risk of bias summary.

Supplement: Supplementary file 14 [file Table_14.DOCX]
